# Supplementary material for: Automated in vivo Tracking of Cortical Oligodendrocytes
Source: Front Cell Neurosci. 2021 Apr 12;15:667595. doi: 10.3389/fncel.2021.667595 (PMC8072161; doi:10.3389/fncel.2021.667595)
Supplement: Supplementary file 1 [file Table_1.DOCX]

**Supplementary Table 1**: Summary of statistical tests and significance level for comparisons by comparison for each referenced figure panel.

| **Figure panel** | **Comparison** | **Statistical test** | **Significance level** |
| --- | --- | --- | --- |
| Figure 7C | Cell volume during recovery over weeks to first significant difference  @ 1 week vs. 2 weeks | Non-parametric Kruskal-Wallis H test with Dunn’s post-hoc test | *p* = < 0.001 |
|  | @ 2 weeks vs. 3 weeks |  | *p* = 0.027 |
|  | @ 3 weeks vs. 4 weeks |  | *p* = 0.79 |
|  | @ 4 weeks vs. 5 weeks |  | *p* = 0.58 |
|  | @ 5 weeks vs. 6 weeks |  | *p* = 0.97 |
|  | @ 6 weeks vs. 7 weeks |  | *p* = 0.25 |
|  | @ 7 weeks vs. 8 weeks |  | *p* = 0.43 |
| Figure 7D | Cell volume of newly formed oligodendrocytes vs. stable control cells  @ 1 week | Non-parametric Kruskal-Wallis H test with Dunn’s post-hoc test and Cohen’s effect size (D) | *p* = < 0.001  *D* = 1.29 |
|  | @ 2 weeks |  | *p* = < 0.001  *D* = 0.85 |
|  | @ 3 weeks |  | *p* = < 0.001  *D* = 0.33 |
|  | @ 4 weeks |  | *p* = 0.39  *D* = 0.07 |
|  | Cell volume of newly formed oligodendrocytes in control timeseries vs. stable control cells  @ 1 week | Non-parametric Kruskal-Wallis H test with Dunn’s post-hoc test and Cohen’s effect size (D) | *p* = < 0.001  *D* = 1.22 |
|  | @ 2 weeks |  | *p* = < 0.001  *D* = 1.10 |
|  | @ 3 weeks |  | *p* = < 0.001  *D* = 0.54 |
|  | @ 4 weeks |  | *p* = 0.28  *D* = 0.09 |
| Figure 7F | Mean distribution of control cell size vs. @ 1 week old cells | 1-way ANOVA with Tukey’s Honest Significant Difference post-hoc test | *p* = < 0.001 |
|  | @ 2 week old cells |  | *p* = < 0.001 |
|  | @ 3 week old cells |  | *p* = < 0.001 |
| Figure 8C | Cell volume during cuprizone treatment over weeks  @ 0 week vs. 1 weeks | Non-parametric Kruskal-Wallis H test with Dunn’s post-hoc test | *p* = < 0.001 |
|  | @ 1 week vs. 2 weeks |  | *p* = < 0.001 |
|  | @ 2 week vs. 3 weeks |  | *p* = < 0.001 |
| Figure 8D | Cell volume of cuprizone treated oligodendrocytes vs. stable control cells  @ 0 week | Non-parametric Kruskal-Wallis H test with Dunn’s post-hoc test and Cohen’s effect size (D) | *p* = 0.001  *D* = 0.30 |
|  | @ 1 week |  | *p* = < 0.001  *D* = 0.26 |
|  | @ 2 weeks |  | *p* = < 0.001  *D* = 0.65 |
|  | @ 3 weeks |  | *p* = < 0.001  *D* = 1.05 |
| Figure 8E | Mean distribution of control cell size vs. cells within 1 week of death | Unpaired 2-tailed t-test and Cohen’s effect size (D) | *p* = < 0.001  *D* = 0*.65* |
